# Role of Exercise Stress Echocardiography in Pulmonary Hypertension

**DOI:** 10.3390/life13061385

**Published:** 2023-06-14

**Authors:** Mojca Škafar, Jana Ambrožič, Janez Toplišek, Marta Cvijić

**Affiliations:** 1 Department of Cardiology, University Medical Centre Ljubljana, Zaloška 2, 1000 Ljubljana, Slovenia; mojca.skafar@gmail.com (M.Š.); jana.ambrozic@gmail.com (J.A.); jtoplisek@gmail.com (J.T.); 2 Faculty of Medicine, University of Ljubljana, Vrazov trg 2, 1000 Ljubljana, Slovenia

**Keywords:** pulmonary hypertension, exercise stress echocardiography, pulmonary arterial pressure, haemodynamics, diagnosis, prognosis

## Abstract

Resting and exercise right heart catheterisation is the gold standard method to diagnose and differentiate types of pulmonary hypertension (PH). As it carries technical challenges, the question arises if non-invasive exercise stress echocardiography may be used as an alternative. Exercise echocardiography can unmask exercise PH, detect the early stages of left ventricular diastolic dysfunction, and, therefore, differentiate between pre- and post-capillary PH. Regardless of the underlying aetiology, a developed PH is associated with increased mortality. Parameters of overt right ventricle (RV) dysfunction, including RV dilation, reduced RV ejection fraction, and elevated right-sided filling pressures, are detectable with resting echocardiography and are associated with worse outcome. However, these measures all fail to identify occult RV dysfunction. Echocardiographic measures of RV contractile reserve during exercise echocardiography are very promising and provide incremental prognostic information on clinical outcome. In this paper, we review pulmonary haemodynamic response to exercise, briefly describe the modalities for assessing pulmonary haemodynamics, and discuss in depth the contemporary key clinical application of exercise stress echocardiography in patients with PH.

## 1. Introduction

Pulmonary hypertension (PH) is a pathophysiological disorder that may involve various clinical conditions and may be associated with numerous respiratory and/or cardiovascular diseases. Regardless of the underlying pathobiological process and the site of the functional changes, PH is defined as a resting mean pulmonary arterial pressure (mPAP) of >20 mmHg measured by right heart catheterisation (RHC) [1].

However, the elevated mPAP is not sufficient to define the subgroups of PH since it could be due to increased pulmonary vascular resistance (PVR) or increased pulmonary artery wedge pressure (PAWP), or just a consequence of alteration in cardiac output (CO) or intrathoracic pressure [1,2]. Taking mPAP, PAWP, and PVR into consideration, we differentiate two main phenotypes of PH. Pre-capillary PH is the consequence of pulmonary vascular disease (e.g., pulmonary artery hypertension, lung diseases, and/or hypoxia, pulmonary artery obstructions,…) and is characterised by the presence of elevated PVR (>2 mmHg/L/min) and normal PAWP (≤15 mmHg). Post-capillary PH is due to left heart disease and is haemodynamically defined as mPAP > 20 mmHg and PAWP > 15 mmHg. The detection and risk stratification of PH patients in everyday clinical practice is often challenging due to the complexity of PH phenotypes; however, it is of paramount importance for adequate treatment.

RHC is the gold standard method to assess pulmonary haemodynamics. It enables diagnosing and classifying PH [1]. On the other hand, echocardiography takes a central role in detecting the consequences of right ventricle (RV) pressure overload, such as RV dysfunction, as well as in detecting the causes of PH, particularly in PH associated with left heart disease. Recent studies have demonstrated that exercise could reveal abnormal pulmonary haemodynamic response in patients with PH, which might provide important prognostic and functional information [3]. According to the current consensus statement, an exercise stress test during RHC is the recommended method to assess cardiopulmonary haemodynamics. However, exercise RHC carries some technical challenges, requiring experienced personnel, special equipment, and additional procedural time. This raises the question of whether non-invasive exercise stress echocardiography may be used as an alternative technique.

The purpose of this manuscript is to review the basic concept of the pulmonary haemodynamic response to exercise in health and disease and to discuss in depth the role of exercise stress echocardiography with a particular emphasis on its diagnostic and prognostic implication in patients with PH.

## 2. How Does Pulmonary Circulation Response to Exercise?

***In healthy subjects:*** An increase in CO during exercise stresses the pulmonary circulation, which results in a physiological increase in pulmonary arterial pressure (PAP). The increase in PAP is strongly dependent on the level of the exercise, and at high exercise levels, it can frequently exceed the mPAP > 20 mmHg [4]. Changes in PAP during exercise are determined by the interplay between CO, pulmonary artery compliance, PVR, and PAWP and can be presented with the following simplified equitation:

mPAP = PVR × CO + PAWP.

**Figure 1:**
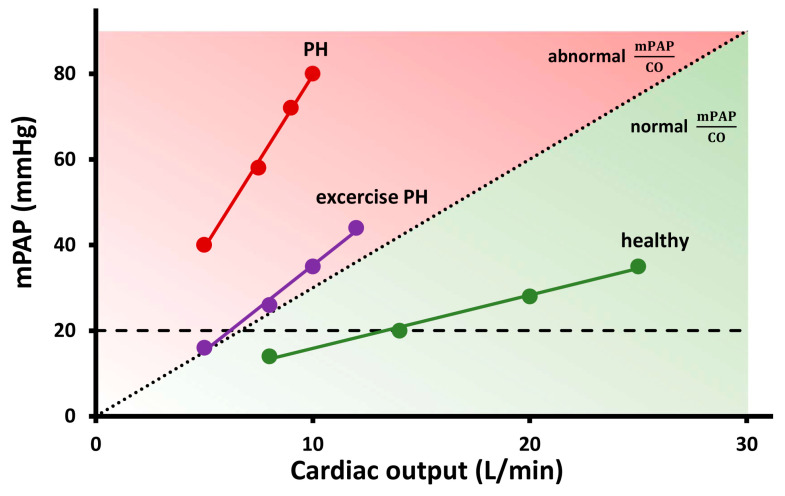
Mean pulmonary arterial pressure (mPAP)–cardiac output (CO) relationship for characterisation of pulmonary haemodynamics during exercise in three representative cases: a patient with pulmonary hypertension (PH), a patient with exercise PH, and a healthy subject. The patient with PH has elevated mPAP at rest and demonstrates disproportionate increase in mPAP during exercise (mPAP/CO = 8 mmHg/L/min), while the patient with exercise, PH has normal resting pulmonary haemodynamics and abnormal response during exercise (mPAP/CO slope = 4 mmHg/L/min). Healthy subject has flow-dependent increase in mPAP, which exceeds 20 mmHg at high CO; however, mPAP/CO slope remains very low (1 mmHg/L/min).

In a normal individual, mPAP and PAWP increase significantly during exercise, while PVR slightly but not significantly decreases [3,5]. The physiological decrease in PVR during exercise is due to the recruitment and distension of the pulmonary resistance vessels with increased flow [6]. The increase in PAP is flow-dependent and needs to be reported in relation to the increase in CO (Figure 1) [7].

In contrast, the mPAP/CO slope is largely unaffected by workload, but it is strongly age dependent. The age dependency of the mPAP/CO slope is mainly determined by the age dependency of the PAWP/CO slope [5]. Higher PAWP/CO slope with ageing is likely due to a decrease in left ventricular (LV) compliance and relaxation during exercise, which is part of the physiological ageing process [8,9]. On the other hand, the distensibility of the pulmonary vessels may remain largely unaffected by age.

***In pathological conditions:*** Unrelated to the pathology, a disproportionate increase in PAP during low levels of exercise is observed, and this contributes to abnormally high mPAP/CO slope (>3 mmHg/L/min) (Figure 1).

In patients with heart failure (post-capillary PH), impaired early diastolic relaxation, reduced increments in suction, and poor LV contractility and/or LV compliance led to inadequate increases in stroke volume and CO with exercise and increased LV filling pressures and PAWP. The high pressure from the left heart is transmitted into the pulmonary circulation, consequently resulting in an increase in mPAP [10,11,12].

On the other hand, patients with pulmonary vascular disease have primarily an increased pulmonary vascular tone and remodelling of the small pulmonary arteries. An abnormal exercise-induced increase in PVR due to pulmonary vascular abnormalities is the most important factor contributing to exercise PAP in this patient’s population [6]. These patients often have reduced CO already at rest and/or fail to adequately augment CO through exercise [13], which results in a steeper mPAP/CO slope during exercise (Figure 1).

A small group of patients have normal pulmonary haemodynamics at rest, but during exercise, a pathological increase in PAP can be detected, evident by an mPAP/CO slope >3 mmHg/L/min between rest and exercise (Figure 1) [5]. The latest evidence demonstrated that this pathological increase in PAP during exercise is associated with a worse prognosis in patients with exercise dyspnoea [14] and several cardiovascular conditions [15,16,17,18]. This entity of exercise PH was recently reintroduced in the 2022 ESC/ERS Guidelines for the diagnosis and treatment of PH as a new phenotype of PH [1].

## 3. Methods for Assessing Pulmonary Haemodynamics

### 3.1. RHC

RHC is the gold standard method to assess pulmonary haemodynamics at rest and during exercise [1]. After confirming PH with mPAP > 20 mmHg at rest, the haemodynamic definition further distinguishes PH into two main phenotypes of PH, pre-capillary and post-capillary PH. PVR is used to differentiate between patients with post-capillary PH who have a significant pre-capillary component (PVR > 2 mmHg/L/min; combined post- and pre-capillary PH) and those who do not (PVR ≤ 2 mmHg/L/min; isolated post-capillary PH) [1]. However, discrimination between subgroups can be challenging if only resting haemodynamics is available, such as when PAWP is close to 15 mmHg or if the clinical characteristics of the patient primarily suggest heart failure with preserved ejection fraction (HFpEF) despite normal resting PAWP [3]. Therefore, the diagnostic work-up requires the use of provocative manoeuvres (e.g., exercise and fluid challenge) during RHC to elicit a dynamic response of the PAWP that may identify the occult post-capillary PH. The latest guidelines recommend using a PAWP cut-off of >25 mmHg during supine exercise for diagnosing post-capillary PH [9]. Although an increased mPAP/CO slope defines an abnormal haemodynamic response to exercise, it does not allow for differentiation between pre- and post-capillary subgroups. However, the PAWP/CO slope with a threshold > 2 mmHg/L/min may best differentiate between pre- and post-capillary causes of exercise PH [19,20].

While the use of resting RHC is widespread, the exercise RHC is a technically demanding diagnostic method as the exercise causes movement artefacts and is available only in specialised centres.

### 3.2. Echocardiography

Echocardiography is a non-invasive, inexpensive, and widely available imaging modality for estimating the systolic pulmonary arterial pressure (sPAP) and detecting additional signs suggestive of PH at rest. However, conventional echocardiography alone is insufficient to confirm a diagnosis of PH as the correlation of sPAP by echocardiography compared with sPAP by RHC was modest, with a correlation coefficient of 0.70 (95% CI 0.67 to 0.73) and with the trends toward underestimation of sPAP by echocardiography [21]. Furthermore, the diagnostic accuracy of echocardiography for PH was also modest, with a sensitivity and specificity of around 80% and 70%, respectively [21,22]. There is no good conventional echocardiographic method that can reliably discriminate between pre- and post-capillary PH at rest. Early studies showed only a modest correlation between the echocardiographic Doppler parameter for the estimation of LV filling pressure (E/e′) at rest and invasively measured PAWP [11]. Similar to the concept of exercise RHC, introducing an exercise test to standard Doppler echocardiography might improve the diagnostic accuracy for detecting post-capillary PH. Furthermore, exercise stress echocardiography has an added value in the management of PH patients, as it also allows us to assess complex RV mechanics (e.g., contractile reserve) and the ability of the pulmonary vasculature to accommodate the increased flow.

Ideally, a semi-supine bicycle test or an upright bicycle exercise protocol with imaging is used [11,12], but there are no universally adopted protocols (Table 1). None of these protocols have been shown to be superior to others [9]. The European Association of Cardiovascular Imaging and the American Society of Echocardiography recommend a stepped protocol on a semi-supine bicycle until the patient reaches his maximal predicted workload and/or maximal predicted heart rate (220—age in years) and/or develops limiting symptoms [12]. However, some patients cannot perform that protocol, and a ramped exercise test has also been proposed for a submaximal target heart rate of 100–110/min or until the patient develops limiting symptoms [23]. There is a paucity of data on isometric exercise, which has little or no effect on CO and may change intrathoracic and systemic arterial pressure and systemic vascular resistance. Therefore, isometric exercise might not be suitable for challenging pulmonary circulation [24].

## 4. Clinical Application of Exercise Stress Echocardiography

### 4.1. Diagnostic Role

In patients with exertional dyspnoea and suspected PH due to HFpEF, there is a possibility to unmask early stages of LV diastolic dysfunction to detect increased LV filling pressures during exercise and, therefore, to differentiate between pre- and post-capillary PH.

As in resting echocardiography, the increase in the E/e′ ratio during exercise is suggestive of elevated LV filling pressures. However, studies comparing haemodynamic data acquired by echocardiography and by RHC during exercise are limited [3,25]. Even though the E/e′ ratio during exercise had only a moderate correlation with directly invasively measured PAWP (r = 0.57; *p* < 0.001), adding the peak exercise E/e′ ratio to the ESC proposed algorithm of diastolic dysfunction improved sensitivity (up to 90%) and can be used to rule out post-capillary PH [10]. Using low-level exercise (20 W) seems to be a good alternative, as E/e′ at 20 W could reliably predict normal PAWP during exercise (AUC: 0.77; *p* < 0.01) [29]. The authors proposed a cut-off of 12.4 for E/e′ at 20 W (specificity 83%, sensitivity 75%). However, these studies comprised only healthy controls and patients with HFpEF. There is only one study that tested the echocardiographic mPAP/CO ratio to identify patients with abnormal pulmonary vascular response to exercise [30]. In a study group of healthy subjects and mainly patients with chronic thromboembolic pulmonary hypertension, mPAP/CO via exercise stress echocardiography of 3.2 mmHg/L/min was identified as the most favourable threshold. Of note, this cut-off is perfectly in line with the proposed cut-off obtained by RHC.

**Figure 2:**
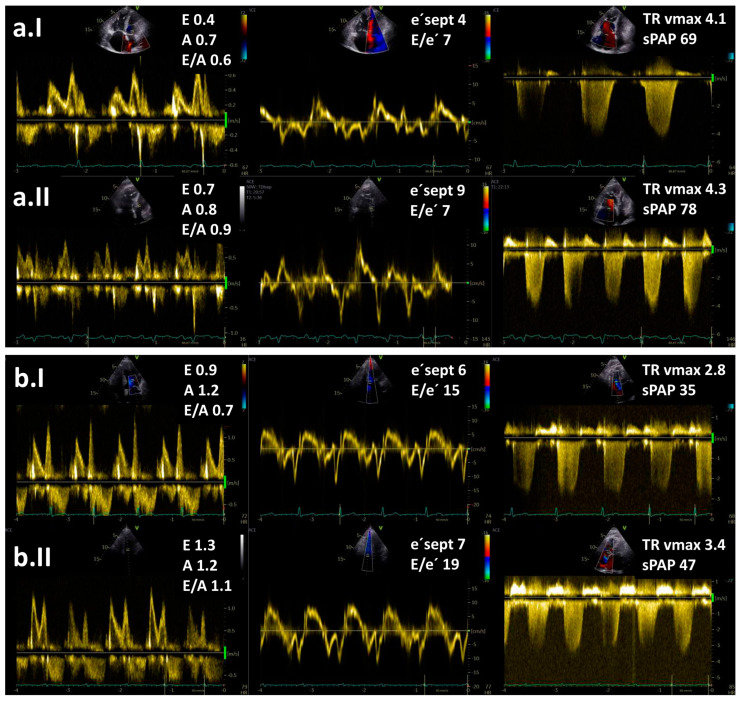
Resting (**I**) and exercise stress echocardiography (**II**) in two patients with pulmonary hypertension (PH): (**a**) a patient with pre-capillary PH and (**b**) a patient with post-capillary PH. Maximal tricuspid regurgitation velocity (TR vmax) is elevated in both patients. Note an increase in E/e′ ratio at low-level exercise from 15 to 19 in a patient with post-capillary PH but no increase in a patient with pre-capillary PH. Legend: sPAP—systolic pulmonary arterial pressure.

In spite of the above-mentioned data, the diagnostic value of the stress tests during echocardiography to distinguish between PH subtypes is currently uncertain due to the lack of prospective data, especially regarding its use to identify cases of combined post- and pre-capillary PH. Based on the data from the literature, exercise echocardiography is considered abnormal if the average E/e′ ratio at peak stress increases to ≥15, with or without a peak TR velocity >3.4 m/s (Figure 2) [10,11,12]. An increase in TR velocity only should not be used to diagnose post-capillary PH because it might be a normal hyperdynamic response to exercise with increased pulmonary blood flow in the absence of LV diastolic dysfunction [31].

Exercise stress echocardiography could be extremely useful as an effective gatekeeper to the RHC for patients with exertional dyspnoea of unknown aetiology and normal resting echocardiographic results and also for identifying patients with a high risk for developing PH. It has been demonstrated that exercise stress echocardiography can distinguish between noncardiac and cardiac causes of unexplained dyspnoea [10,32]. The diagnostic value of exercise stress echocardiography was also evaluated in asymptomatic relatives of patients with idiopathic and familial PAH [33]. Hypertensive response to exercise, defined by TR velocity > 3.1 m/s, was more often present in relatives of PAH patients than in control subjects. Additionally, exercise stress echocardiography is considered to be reasonable, especially in patients with connective tissue disease [34]. It has been reported that up to 50% of this patient population with normal resting mPAP had an abnormal increase in mPAP during exercise. In patients with systemic sclerosis, PH was confirmed by RHC in 81% of patients with positive exercise stress echocardiography [35]. Moreover, exercise stress echocardiography could unmask exercise PH in patients with systemic sclerosis and baseline echocardiographic PAP within the grey zone [36]. It is important to note that in both studies, authors used a definition of exercise PH, which is not in line with nowadays valid definition (an increase of 20 mmHg over the resting sPAP or sPAP > 50 mmHg was considered as a positive test result). However, the clinical value of exercise PH identified by exercise stress echocardiography remains uncertain because of the lack of validated criteria and prospective confirmatory data [1]. Therefore, data from exercise stress echocardiography are not sufficient to be a substitute for invasive haemodynamic data under all circumstances, especially if a therapeutic decision depends on the results [9].

### 4.2. Prognostic Role

Regardless of the underlying aetiology, the developed PH is associated with worsening symptoms and substantially increased mortality [37]. Even though the detection of exercise PH via exercise stress echocardiography is considered an early and mild phase of PAH [38], patients with exercise PH already had worse outcomes than subjects without exercise PH [39].

The survival of PH patients depends on the capability of the RV to adapt to chronically elevated PAP [40]. Over time, adaptive concentric RV hypertrophy with preserved RV function can evolve into RV dilatation and systolic dysfunction [41,42]. RV function is a major determinant of functional capacity and prognosis when RV afterload is elevated [43,44,45]. Echocardiographic measures of RV function that are independent predictors of mortality in PH include the tricuspid annular plane systolic excursion (TAPSE < 18 mm [46,47,48]), RV fractional area change (FAC < 35% [49,50]), peak systolic tricuspid lateral annular velocity (S’ < 9.7 cm/s [51]) and Tei index (>0.40 by pulse Doppler or >0.55 by tissue Doppler [52]). Conventional 2-dimensional echocardiographic evaluation of the RV is difficult due to the complex 3-dimensional (3D) anatomical shape of the RV. This limitation can be overcome with 3D echocardiography and/or cardiac magnetic resonance [53,54] and recently, an increased 3D RVESVi has been shown to correlate with increased mortality [55].

However, these parameters all fail to identify occult RV dysfunction in patients with PH [56] as they reflect already established RV dysfunction. Subtle RV dysfunction could possibly be recognised by the use of advanced echocardiographic techniques, such as strain/myocardial deformation and myocardial work [57,58,59]. Previous studies demonstrated that RV longitudinal strain was a powerful predictor of survival in patients with PH and provided incremental prognostic value over conventional clinical and echocardiographic variables [60,61].

Additionally, the assessment of RV contractile reserve via RV–pulmonary arterial (PA) coupling shows promising results in detecting subclinical RV systolic dysfunction [56,62,63,64,65,66]. Gold standard measurement of RV–PA coupling involves conductance catheter measurement of “multi-beat” RV end-systolic elastance (Ees), a method that remains costly, impractical and clinically challenging [62]. However, new echocardiographic indices, such as the TAPSE/sPAP ratio [45,67,68,69] and RV free wall longitudinal strain/sPAP [59], are tightly linked to RV–PA coupling and are associated with outcomes in patients with PH.

A possible non-invasive measure of the RV contractile reserve using exercise stress echocardiography was first proposed by Grünig et al. They demonstrated that an exercise-induced increase in sPAP was a measure of the RV contractile reserve and was an independent prognostic factor in patients with pre-capillary PH (Table 1) [70]. A lower sPAP increase may reveal an impaired ability of the RV to adapt to pulmonary load and exercise and to further increase pressure and pulmonary blood flow. Similarly, an initial steep increment in PAP during exercise followed by a plateau with a linear pattern was associated with decreased exercise capacity and survival in patients with heart failure [17]. Echocardiographic studies focused only on the peak exercise sPAP or the peak change in sPAP [17,70]; however, it would be preferable to interpret exercise PAP pattern relative to the increase in blood flow (PAP/CO ratio). Invasively obtained haemodynamic data clearly showed that high mPAP/CO during exercise was associated with impaired survival in a heterogeneous group of different PH phenotypes [5,14]. Echocardiographic studies analysing mPAP/CO are limited, but initial results are very promising. A disproportionate increase in mPAP/CO slope during exercise was independently associated with adverse clinical outcomes in patients with HFpEF (Table 2) [71], and this parameter had an incremental value even in patients with preserved RV-PA coupling at rest.

Other authors assessed RV contractile reserve based on echocardiographic parameters of RV systolic function (e.g., change in TAPSE, change in RV FAC and change in S’) (Table 2) [72,73]. The magnitude of the increase in all three parameters was significantly lower in patients with pre-capillary PH than in healthy controls [72,73]; however, no prognostic data have been available for these parameters. Ireland et al. prospectively studied RV contractile reserve in PH patients who underwent cardiac magnetic resonance, echocardiography, and supine invasive cardiopulmonary exercise testing with concomitant RV pressure-volume catheterisation. RV contractile reserve during exercise, measured by Ees during exertion, was associated with an elevation in PAP but the preservation of RV volumes. The lack of RV reserve, on the other hand, was tightly coupled with acute RV dilation during exercise [62]. RV ejection fraction during exercise was shown to be a robust surrogate for RV contractile reserve (Table 2), and it best predicted occult RV dysfunction among a variety of resting and exercise RV measures and was also associated with clinical worsening [62]. Therefore, echocardiographic parameters of RV contractile reserve and exercise stress echocardiography could be useful for follow-up assessment, especially to identify PH patients at high risk [70].

**Table 2:** Non-invasive measures of the right ventricle (RV) contractile reserve during exercise stress echocardiography.

Author	Subjects (n)	Echocardiographic Parameters	Most Relevant Findings
Grünig, 2013 [70]	124 PH patients (PAH, CTEPH) and impaired RV systolic function	∆sPAP	Exercise-induced sPAP increase ≤ 30 mmHg was related to the worst outcome (HR 2.84, 95% CI 1.92–6.78; *p* = 0.018).
Almeida, 2014 [73]	14 subjects (7 controls, 7 patients with PH)	∆S’, ∆TAPSE and ∆FAC	The magnitude of increase in ∆S’, ∆TAPSE and ∆FAC in healthy controls was higher than in patients (all *p* < 0.05.
Guo, 2019 [72]	46 subjects (31 patients with pre-capillary PH, 15 controls)	∆S’, ∆TAPSE and ∆FAC	Significant increase in ∆S’ (*p* = 0.002), ∆TAPSE (*p* < 0.001) and ∆FAC (*p* < 0.001) was noted only in healthy controls.
Ireland, 2021 [62]	35 subjects with known or suspected PH	Exercise RVEF	Exercise RVEF can detect occult RV dysfunction (AUC = 0.81, cut off of exercise RVEF = 38%). Patients with exercise RVEF < 38% had an increased propensity for clinical worsening over 4 years compared to patients with RVEF > 38% (*p* = 0.014).
Saito, 2023 [71]	345 patients (1666 HFpEF, 179 controls)	mPAP/CO slope	Patients with mPAP/CO slope > 5.2 mmHg/L/min had a higher rate of adverse events (all-cause mortality, HF events) compared to those with mPAP/CO slope < 5.2 mmHg/L/min (*p* = 0.0002).

Legend: CO—cardiac output, CTEPH—chronic thromboembolic pulmonary hypertension, FAC—fractional area change, HFpEF—heart failure with preserved ejection fraction, mPAP—mean pulmonary arterial pressure, PAH—pulmonary arterial hypertension, PH—pulmonary hypertension, RVEF—right ventricular ejection fraction, sPAP—systolic pulmonary arterial pressure, S’ -Doppler-derived tricuspid lateral annular peak systolic velocity, and TAPSE—tricuspid annular plane systolic excursion.

### 4.3. Practical Approach to Exercise Stress Echocardiography

Detailed practical guidelines on stress echocardiography have already been published [74]. For the purpose of this review, only some important factors are emphasised:

- In the case of a step protocol, haemodynamic measurements are performed towards the end of each exercise level when a steady state in oxygen consumption on a given exercise level is achieved (usually in 3–5 min). For practical reasons, shorter time intervals can be chosen (e.g., 2 min steps aiming for a duration of the exercise time of∼10 min), which appear to be a good compromise [3].
- The feasibility of obtaining diagnostic-quality measurements of TR velocity decreases with increasing exercise load, with 54% at low exercise (20 W) and 49% at peak exercise [10]. The administration of agitated colloids enhances a continuous Doppler tricuspid regurgitation signal and allows reliable estimation of sPAP during exercise [30].
- At higher heart rates, the fusion of the mitral E and A waves prevents the estimation of the LV filling pressures. It was reported that E/e′ could not be measured in about 10% of subjects during submaximal exercise (20 W) and in about 25% of patients during peak exercise [29]. Therefore, the acquisition of images during the submaximal phase before the fusion of E and A waves is advised (heart rate 100–110 bpm) [75].
- Acquisition during early recovery is not optimal, as haemodynamics change very rapidly after cessation of exercise, and previous invasive studies demonstrated that PAWP returned to the baseline levels already 1 min post-exercise [76].

## 5. Future Perspectives

Many different protocols of exercise stress echocardiography are used in everyday clinical practice, and further research is needed to compare these protocols and possibly universally adopt one of them. The diagnosis of exercise PH, which was reintroduced in the newest ESC Guidelines for the diagnosis and treatment of PH, is challenging [1]. It requires exercise RHC, which is not readily available. Further research is needed to determine non-invasive cut-offs that would reveal possibly abnormal exercise pulmonary haemodynamics. Moreover, there is no reliable non-invasive method for discrimination between pre-capillary and post-capillary PH in dubious cases. There are some non-invasive stress tests that could be used in such cases to avoid invasive procedures, but the data about their usability are limited. RV dysfunction is the most important determinant of survival in patients with PH [43,44]. However, the manifestations of RV dysfunction not only include changes in global RV systolic function but also abnormalities in the pattern of contraction and synchrony that can be analysed using RV strain and RV myocardial work. The use of these novel parameters for prognostic assessment of PH patients is relatively new and mainly used for research purposes [57,58,59]. Further data are needed to implement them into exercise stress echocardiography in PH patients.

## 6. Conclusions

Exercise stress echocardiography is a promising non-invasive tool for the assessment of pulmonary haemodynamics, as it provides clinically relevant diagnostic and prognostic information in patients with PH. However, studies comparing echocardiography and RHC data during exercise are limited, and there is no single echocardiographic parameter that reliably identifies the underlying aetiology of PH. Therefore, RHC remains the gold standard method for the diagnosis and classification of different types of PH. In contrast to RHC, echocardiography has the ability to identify RV remodelling and systolic dysfunction, which are important determinants of survival in patients with PH. However, more research is needed since there are still many different protocols of exercise stress echocardiography used in everyday clinical practice, and many new promising echocardiographic parameters for the prognostic assessment of PH patients are emerging.

## Figures and Tables

**Table 1:** Commonly used exercise stress echocardiography protocols currently employed in clinical practice.

Author	Type	Protocol
Ha, 2020 [25]	semi-supine bicycle	stepped protocol: cycling at a cadence of 60 r.p.m. starting at 25 W and increasing in increments of 25 W every 3 min
Erdei, 2014 [23]	semi-supine bicycle	ramp protocol: cycling at a cadence of 60 r.p.m. starting at 15 W with 5 W increments every minute
Motram, 2004 [26]	treadmill	Bruce protocol
Jake Samuel, 2017 [27]	isometric handgrip	holding the dynamometer at 40% of MVC for 3 min
Pongpaopattanakul, 2022 [28]	dynamic handgrip	squeezing the dynamometer at 2 kg at a cadence of 30 r.p.m. for 3 min

Legend: MVC—maximal voluntary contraction; r.p.m.—revolutions per minute.

## Data Availability

Data sharing not applicable.
